# A new species of *Espeletiopsis* (Millerieae, Asteraceae) from Colombia

**DOI:** 10.3897/phytokeys.32.6387

**Published:** 2013-12-19

**Authors:** Mauricio Diazgranados, Luis Roberto Sánchez

**Affiliations:** 1 Dept. of Botany, MRC 166, National Museum of Natural History, P.O. Box 37012, Smithsonian Institution, Washington D.C. 20013-7012, United States; 2 Departamento de Biología y Química. Universidad de Pamplona. Pamplona, Colombia

**Keywords:** Cáchira, Colombia, Compositae, Espeletiinae, *Espeletiopsis*, frailejón, Millerieae, Norte de Santander, páramos

## Abstract

A new species of *Espeletiopsis* was found in two small páramos of Norte de Santander, Colombia. The species is named *Espeletiopsis diazii* honoring the contributions of Santiago Díaz-Piedrahita in recognition of his vast knowledge of the Compositae in Colombia. This is a very distinctive species, markedly different from most of the *Espeletiopsis* present in Colombia. The new species is closely related to *Espeletiopsis caldasii* and *Espeletiopsis santanderensis*, but differs in having (1–)4–6(–7) capitula, with very short peduncles, and capitula arranged in a compact or densely glomerate cyme. With a total distribution area of less than 75 km^2^, this species is probably critically endangered or imperiled.

## Introduction

At the high elevations of the tropical Andes, typically above 3600 m, trees and shrubs disappear gradually, opening the space for the páramo. With an estimated age of 2–4 million years (Van der Hammen et al. 1986), the páramo ecosystem is relatively young and yet is widely regarded as the world’s most diverse high-elevation ecosystem (Luteyn 1999; Rangel-Ch. 2000; Sklenář et al. 2005), and probably the fastest evolving biodiversity hotspot (Madriñán et al. 2013). One of the unique characteristics of the páramo is the presence of frailejones, the name being generally used to refer to all the species within the subtribe Espeletiinae Cuatrec. (Asteraceae: Millerieae).

The subtribe Espeletiinae Cuatrec. (Millerieae, Asteraceae) includes eight genera and at least 141 species (Cuatrecasas 2013; Diazgranados 2012a). In the monograph of Espeletiinae, Cuatrecasas (2013) treated seven of these genera in detail, but *Espeletiopsis* remained untreated. This genus is diagnosed by axillary, monochasial, corymbiform-paniculate capitulescences, with branches, leaves and bracts spirally alternate, and involucres with gradual transition from sterile outer phyllaries to fertile inner phyllaries (Cuatrecasas 1976). Currently the genus contains 22 species, two varieties, three forms and three described hybrids (Diazgranados 2012a).

The genus *Espeletiopsis* is distributed from the central Andes of Venezuela to the Cordillera Oriental (Eastern Cordillera) in Colombia, approximately from 70.8°W, 8.9°N to 74.3°W, 4.1°N). Only five species are found in Venezuela (four of them endemic to the country), whereas Colombia has 18 species. According to Cuatrecasas (2013), *Espeletiopsis* represents a derived line of evolution within Espeletiinae, being mainly adapted to lower elevations. Twenty species are found at elevations of 3200–3400 m in the subpáramo belt, two species grow as low as 2200 m, and one reaches 4500 m.

Most of the species are restricted to one continuous páramo and have relatively narrow distributions (<1000 km^2^). Seven species have distributions of 2500–4750 km^2^, six species are distributed in areas of less than 20 km^2^, and six species are known from less than seven collections, including two with only the type collection (Diazgranados 2012a; b). In the last ten years four new species of *Espeletiopsis* have been described from Colombia (Díaz-Piedrahita and Obando 2004; Díaz-Piedrahita and Rodriguez-Cabeza 2008; 2010; Díaz-Piedrahita et al. 2006), and it is not surprising that more new species will be discovered in uncollected páramos.

Species of *Espeletiopsis* have not been studied thoroughly. In addition to general morphology and reports in inventories and floras, six species have studies on anatomy (Carlquist 1958; Ortega 1982; Rock 1972; Torres de Ricardi 1979), six on chemistry (Bohlmann et al. 1980; Ramírez et al. 1998; Ramírez et al. 2000; Usubillaga et al. 2003; Meccia et al. 2010), two on physiology (Bayona et al. 2005; Coba de Gutiérrez 2005; Rache and Pacheco 2009), one on demography (Chaves and Bonilla 2007), one in ethnobotany (Báez et al. 1999), three in animal-plant interactions (Berry and Calvo 1989; Sturm 1989; 1990a, b), one in mycorrhizae (García Romero et al. 2004) and 12 have chromosome counts (Cuatrecasas 2013; Rondón et al. 2013). The most studied species are *Espeletiopsis corymbosa* (Humb. & Bonpl.) Cuatrec., *Espeletiopsis muiska* (Cuatrec.) Cuatrec., *Espeletiopsis angustifolia* (Cuatrec.) Cuatrec. and *Espeletiopsis pannosa* (Standl.) Cuatrec., all relatively close to major cities in Colombia and Venezuela. Several species in more pristine páramos remain largely unstudied. Unfortunately, páramos are shrinking rapidly as a consequence of climate change, and several species are predicted to become extinct in the following decades (Diazgranados 2012b).

## Methods

The Páramo de Cáchira (also called Páramo de Guerrero; see Fig. 4) and the adjacent Páramo de los Ranchos are located in a region of Norte de Santander (Colombia) of very difficult access. North from the Páramo de Arboledas, these are probably the last páramos of the Eastern Cordillera before the Ocaña depression. For decades the area was unsafe, and the only road that reaches these páramos was almost impassable. Still now, the area is floristically poorly known. Material of the new species was collected during an expedition in 2009, and duplicates distributed to COL, ANDES and HECASA. Additional duplicates will be distributed to other herbaria, including US and MO. Micrographs were taken by the first author at the Scanning Electron Microscopy Laboratory of the National Museum of Natural History, in Washington DC. Lauren Merchant from Saint Louis University provided the illustrations, which were funded by the Missouri Botanical Garden.

## Taxonomy

### Espeletiopsis diazii

M. Diazgranados & L.R. Sánchez sp. nov.

urn:lsid:ipni.org:names:77134808-1

http://species-id.net/wiki/Espeletiopsis_diazii

**Figure 1.**
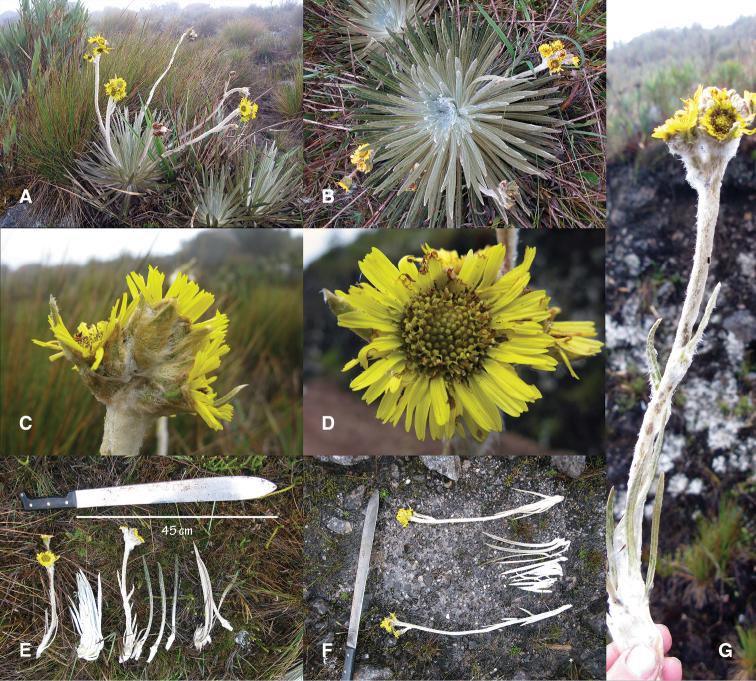
*Espeletiopsis diazii*:**A** acaulescent (sessile) habit **B** whitish rosette from top **C** lateral view of capitulescence showing a dense glomerate cyme of capitula **D** capitulum of the holotype collection (*M. Diazgranados* & *L.R. Sánchez 3898*) **E** paratype collection (*M. Diazgranados & L.R. Sánchez 3897*) **F** holotype collection (*M. Diazgranados & L.R. Sánchez 3898*) **G** capitulescence showing the alternate bracts along the scape.

Figures 1 [Fig F2] [Fig F3] –4

#### Type.

COLOMBIA, Norte de Santander, Municipio de Cáchira, Páramo de Cáchira o de Guerrero, vía Alto Chiquito (desde Villa Caro) a Cáchira, alto del páramo, a los lados de la vía antes de comenzar el descenso a Cáchira, en comunidad de pajonal-frailejonal dominado por esta especie. Alt. 3394 m, 73.00173°W, 7.7655°N. *M. Diazgranados* & *L.R. Sánchez 3898* (holotype: COL; isotypes: COL, HECASA and to be distributed). Paratypes: same locality, *M. Diazgranados* & *L.R. Sánchez 3897* (COL, HECASA and to be distributed); *L.R. Sánchez 10113* (HECASA!), *12714* (HECASA!).

#### Diagnosis.

Sessile rosette of whitish appearance, related to *Espeletiopsis santanderensis*, but with smaller leaves, capitulescence compact with a dense glomerate cyme of (1–)4–6(–7) capitula and short peduncles, and disc flowers with corolla lobes glabrescent.

#### Description.

Acaulescent (sessile) polycarpic rosette of whitish appearance, 40–50(–100) cm tall (including capitulescences), growing in grassland of páramo proper. Excluding reproductive parts, rosette 20–30(–50) cm tall.

Leaves firm, coriaceous, erect; laminae linear or narrowly oblanceolate, apex acute, base without pseudopetiole, (15–)16–19(–21) cm × (0.6–)0.65–0.7(–0.8) cm, length to width ratio 26:1; margins strongly revolute. Adaxial face with indumentum whitish or silvery-sericeous, hairs 1 mm long, very abundant in young leaves, becoming less dense and almost tomentose-velutinous in old leaves, giving them a greener appearance; costa prominent but secondary nerves invisible. Abaxial face with loose indumentum, silvery or whitish, with hairs up to 2 mm long; costa more prominent than from adaxial face, secondary nerves invisible. Leaf sheaths narrowly-oblong, (2.5–)2.7–3.1(–3.3) cm × (0.5–)0.7–0.9(–1.1) cm; white adaxially, glabrescent in the proximal portion, with 10–15 anastomosing nerves; white abaxially, tomentose-velutinous, with hairs up to 0.9 mm long.

Capitulescences 2–5, corymbiform, more or less coetaneous, axillary, emerging from the upper nodes, twice longer than the leaves, (15–)25–45(–60) cm long; indumentum abundant, lanose-sericeous white towards the base, becoming lanose whitish-yellowish towards the distal portion. Scapes erect, firm, 5–6 mm in diameter; 5–10-bracteate basally, subcoriaceous, alternate, linear with acute apex and without pseudopetiole, up to 13 cm long × 0.5 cm wide; 2–5 sterile bracts in the first 2/3 of the scape, alternate, linear, shorter, 5–6 cm long × 0.5 cm wide. Capitula (1–)4–6(–7), arranged in a compact or dense glomerate cyme in the distal 3–4 cm of the capitulescence; peduncles terete, short, 1(–3) cm long or less; with indumentum lanose whitish-yellowish, and portions of epidermis reddish. Fertile bracts linear or narrowly triangular, 2–2.5 cm long × 0.4–0.5 cm wide.

Capitula radiate, subglobose, 2.0–3.0 cm in diameter (including ray flowers). Involucre 1.2–1.4 cm wide × 0.8–1.2 cm high; phyllaries in 2–3 series, oblanceolate or triangular, the outer phyllaries 10–12 mm long × 3.0–5.0 mm wide (excluding hairs), the inner phyllaries 6.5–7.2 mm long × 4.0–4.3 mm wide, with indumentum villous white and epidermis green, turning red when older.

Ray flowers (30–)44–46 in 2(–3) series, yellow, 10.5–11.5 mm long (excluding ovary). Ligules 7.6–8.0 mm long, elliptical or oblong, tridentate; tube hirsute, small, 0.35–0.5 mm in diameter and 2.5–3.0 mm long, without linguiform appendages, yellow becoming brown distally, the hairs 0.2–0.3(–0.7) mm long. Style 6.5–7.0 mm long × 0.16–0.2 mm in diameter, with stigmatic branches 1.0–1.5 mm long, broadening in the distal portion, 0.25–0.28(–0.5) mm wide, papillose, papillae to 0.1 mm long.

Discs 1.0–1.5 cm in diameter; disc flowers (72–)100–108; corolla 6.2–7 mm long (excluding anthers and fruit); corolla throat 4.4–4.6 mm long, 2–2.2 mm wide when open, 5-lobed, lobes 1–1.1 mm long, glabrescent or with a few hairs; tube 1.8–2.4 mm long × 0.3–0.5 mm in diameter, glabrous, with a few hairs; anthers dark yellow, sometimes exceeding the corolla by 2 mm, slightly translucid, approximately 1 mm long and 0.3 mm wide; disc paleae 5.2–5.5 mm long × 1.0–1.1 mm wide, brownish, with 3 main nerves, glabrescent but becoming villous in the distal third.

Cypselae oblong, triangular, 2.3–2.4 mm × 1.5–1.6 mm, glabrous, black. Paleae 6.4–6.8 mm long, 1.8–2.0 mm wide, brownish, profusely villous. Pollen yellow when fresh, tricolporate, 20.56–21.08 μm in equatorial diameter (not counting spines); spines 68–80 total, 14–16 equatorial spines, (2.8–)3.6–4.06 μm long, erect.

**Figure 2. F2:**
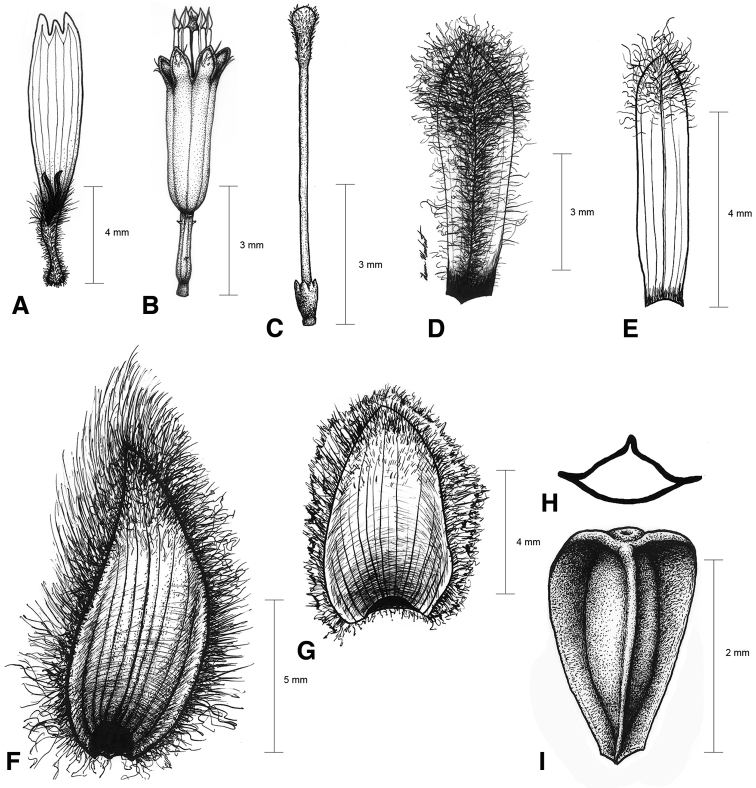
Illustrations of *Espeletiopsis diazii*. **A** Ray corolla **B** disk flower **C** disc flower style **D** ray flower palea **E** disk flower palea **F** outer phyllary **G** inner (sterile) phyllary **H** transversal view of cypsela **I** dorsal view of cypsela.

**Figure 3. F3:**
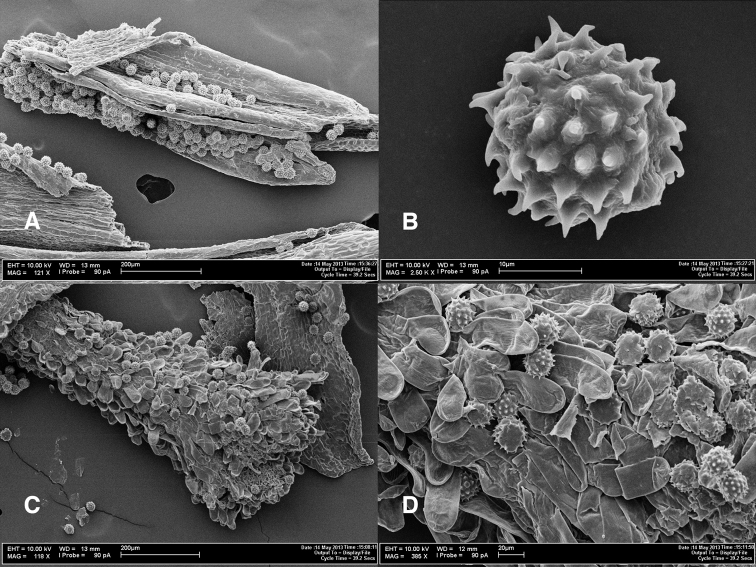
Photomicrographs of *Espeletiopsis diazii*. **A** Fragment of anther with pollen grains **B** pollen grain **C** disc flower style with pollen grains **D** papillae of outer side of stigmatic branches, showing pollen grains.

#### Distribution.

**Figure 4.**
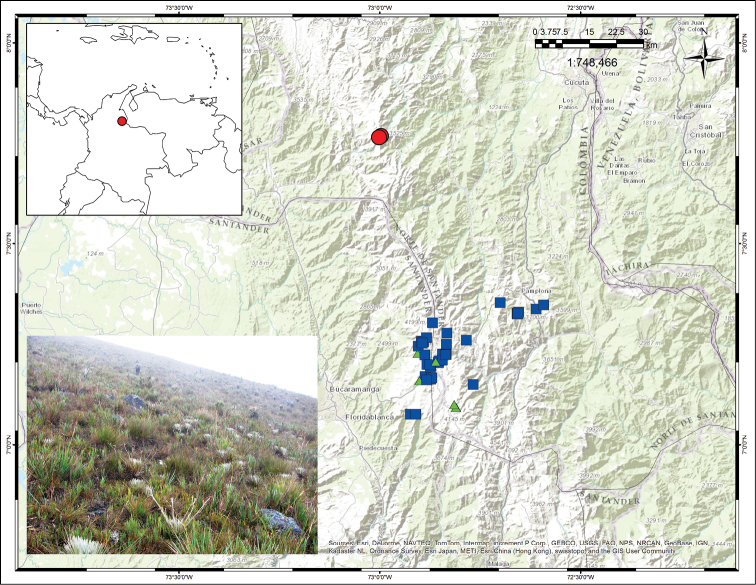
Distribution map showing collections for *Espeletiopsis diazii* (red circles), *Espeletiopsis santanderensis* (blue squares) and *Espeletiopsis caldasii* (green triangles). Photograph of the Páramo de Cáchira, with a population of *Espeletiopsis diazii*.

Endemic to Colombia. This species has been found only in the Páramo de Cáchira (or Páramo de Guerrero), and in a smaller adjacent páramo, called Páramo de los Ranchos, at elevations of 3300–3500 m (Fig. 4). The area of distribution is less than 75 km^2^.

#### Ecology.

A large population of several hundreds or thousands of individuals growing in the grasslands of the páramo proper was observed (Fig. 4). Other Espeletiinae found in the area are: *Espeletia brassicoidea* Cuatrec., *Espeletiopsis conglomerata* Cuatrec. and *Libanothamnus occultus* ssp. *oroquensis* Cuatrec.

#### Etymology.

The specific epithet of this new species, “diazii”, is dedicated to Santiago Díaz-Piedrahita, Colombian botanist, for his vast contributions to the knowledge of the Compositae of his country.

#### Conservation status.

The preservation of this species is linked to the preservation of the Páramo de Cáchira and the Páramo de Los Ranchos. These are both very small páramos (70–80 km^2^ of total area), likely sensitive to climate and land use change, with substantial fragmentation, and without any legal measures of protection. Therefore, *Espeletiopsis diazii* is probably *Critically Endangered* (CR, according to the IUCN criteria: extent of occurrence estimated to be less than 100 km^2^, habitat fragmentation, and likely decline of the extent of the páramo; http://jr.iucnredlist.org/documents/redlist_cats_crit_en.pdf ), or *Critically Imperiled* (G1, according to NatureServe; http://www.natureserve.org/explorer/ranking.htm ).

## Discussion

*Espeletiopsis diazii* is a very distinctive species. The laminae lack pseudopetioles and the sheaths are oblong, rectangular, not broadening toward the base, the features being rare in *Espeletiopsis* but characteristic of *Espeletia* sect. *Weddellia* Cuatrec. (8 species) of the páramos of Apartaderos, Piñango and Timotes in Mérida, Trujillo and Lara, Venezuela. However, monochasial capitulescences with several alternate bracts along the scape place this species within *Espeletiopsis*.

**Figure 5.**
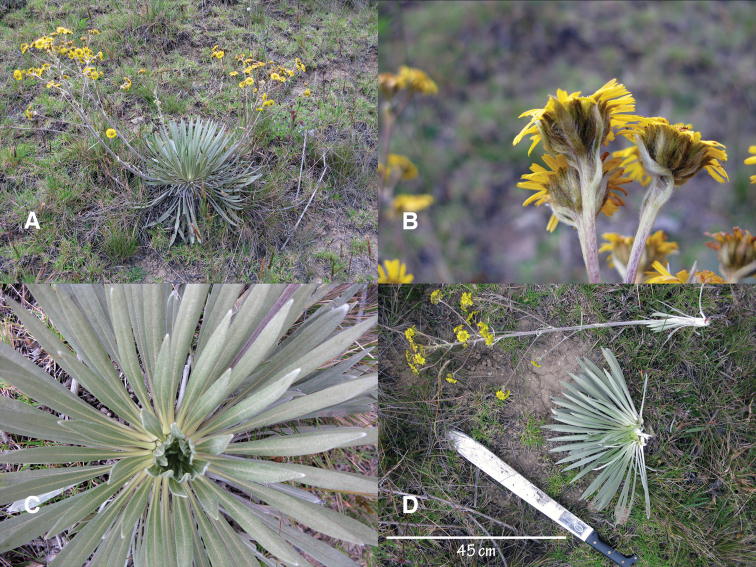
*Espeletiopsis santanderensis*:**A** acaulescent (sessile) habit **B** lateral view of a section of the capitulescence, showing the long peduncles of the capitula **C** rosette from the top **D** capitulescence spreading and rosette (*M. Diazgranados 3841*).

The new species is probably related to a group of 10 species of *Espeletiopsis* with sessile caulirosula habit (sensu Cuatrecasas 2013) from the páramos. Two of these (*Espeletiopsis angustifolia* (Cuatrec.) Cuatrec. and *Espeletiopsis pannosa* (Standl.) Cuatrec.) also have an indumentum that is silvery-sericeous, but differ from the rest in having white-purple ligulae. The remaining eight species, all from Colombia, have yellow ligulae: *Espeletiopsis betancurii* Rodr.-Cabeza, S. Díaz & Gal.-Tar., *Espeletiopsis caldasii* (Cuatrec.) Cuatrec., *Espeletiopsis colombiana* (Cuatrec.) Cuatrec., *Espeletiopsis funckii* (Sch. Bip. ex Wedd.) Cuatrec., *Espeletiopsis muiska* (Cuatrec.) Cuatrec., *Espeletiopsis petiolata* (Cuatrec.) Cuatrec., *Espeletiopsis pozoensis* (Cuatrec.) Cuatrec., and *Espeletiopsis santanderensis* (A. C. Sm.) Cuatrec. However, only two of them (i.e., *Espeletiopsis santanderensis* and *Espeletiopsis caldasii*) have a reduced silvery-sericeous indumentum and reduced oblong sheaths. *Espeletiopsis caldasii* has capitulescences monocephalous, and *Espeletiopsis santanderensis* has capitulescences spreading (Fig. 5). Both species grow in the páramos of Santander-Norte de Santander, but relatively distantly from the population of *Espeletiopsis diazii* (Fig. 4).

### Key to *Espeletiopsis diazii*, *Espeletiopsis caldasii* and *Espeletiopsis santanderensis*

A key for the genus was initially published by Cuatrecasas (1996) and updated later by Díaz-Piedrahita and Rodríguez-Cabeza (2010). This key is an extension of the updated version of Cuatrecasas’ key.

**Table:** 

4	Leaf lamina up to 25 cm long × 0.3–1.5 cm wide, with indumentum dense, appressed, silvery-sericeous.
5	Leaf lamina 4–6 cm long × 0.3–0.9 cm wide. Capitulescence monocephalous	*Espeletiopsis caldasii*
5’	Leaf lamina 15–25 cm long × 0.6–1.5 cm wide. Capitulescence polycephalous.
6	Capitulescence spreading, of 15–20 cm in diameter, with 20–40 capitula, peduncles 3–7 cm long. Disc flowers with corolla lobes ± pilose. Leaf lamina 15–25 cm long × 0.8–1.5 cm wide	*Espeletiopsis santanderensis*
6’	Capitulescence compact, with a dense glomerate cyme of (1–)4–6(–7) capitula, peduncles up to 1(–3) cm long. Disc flowers with corolla lobes glabrescent or with a few hairs. Leaf lamina (15–)16–19(–21) cm long × (0.6–)0.65–0.7 (–0.8) cm wide	*Espeletiopsis diazii*

## Supplementary Material

XML Treatment for Espeletiopsis diazii
